# Area under the inspiratory flow-volume curve (AIN): Proposed normative values

**DOI:** 10.1371/journal.pone.0307966

**Published:** 2024-08-01

**Authors:** Octavian C. Ioachimescu, James K. Stoller

**Affiliations:** 1 Professor of Medicine, Division of Pulmonary, Critical Care and Sleep Medicine, Department of Medicine and Clinical and Translational Science Institute, Medical College of Wisconsin, Milwaukee, Wisconsin, United States of America; 2 Adjunct Professor of Medicine, Division of Pulmonary, Allergy, Critical Care and Sleep Medicine, Department of Medicine, School of Medicine, Emory University, Atlanta, Georgia, United States of America; 3 Staff Physician, Clement J. Zablocki Veteran Affairs Medical Center, Milwaukee, Wisconsin, United States of America; 4 Jean Wall Bennett Professor of Medicine and Chair, Education Institute, Cleveland Clinic, Cleveland, Ohio, United States of America; All India Institute of Medical Sciences - Raipur, INDIA

## Abstract

**Rationale:**

Area under expiratory flow-volume curve (AEX) has been shown to be a valuable functional measurement in respiratory physiology. Area under inspiratory flow-volume loop (AIN) also shows promise in characterizing upper and/or lower airflow obstruction.

**Objectives:**

we aimed here to develop normative reference values for AIN, able to ascertain deviations from normal.

**Methods:**

We analyzed AIN in 4,980 spirometry tests recorded in non-smoking, healthy individuals in the Pulmonary Function Testing Laboratory.

**Results:**

The mean (95% confidence interval, CI), standard deviation and median (25^th^-75^th^ interquartile range) AIN were 16.05 (15.79–16.31), 9.08 and 14.72 (9.12–21.42) L^2^·sec^-1^, respectively. The mean (95% CI) and standard deviation of the best-trial measurements for square root of AIN (Sqrt AIN) were 3.84 (3.81–3.87) and 1.14; 4.15 (4.12–4.18) and 1.03 in men, and 2.68 (2.63–2.72) and 0.72 L·sec^-1/2^ in women. The mean (standard deviation) of pre- and post-bronchodilator Sqrt AIN were 3.71 (1.17) and 3.81 (1.19) L·sec^-1/2^, respectively.

The mean (95% CI), standard deviation and lowest 5^th^ percentile (lower limit of normal, LLN) of Sqrt AIN/Sqrt AEX (%) were 101.3 (100.82–101.88), 18.7, and 71.8%; stratified by gender, it was 102.2 (101.6–102.8), 18.6, and 72.8% in men, and 98 (96.9–99.2), 18.8, and 68.6% in women, respectively.

**Conclusions:**

The availability of area under the inspiratory flow-volume curve (AIN) and the derived indices offers a promising opportunity to assess upper airway disease (e.g., involvement of larynx, trachea or major bronchi), especially because some of these measurements appear to be independent of age, race, height, and weight.

## Introduction

In 1667, Robert Hooke managed to keep a dog alive by artificial respiration using bellows, proving the vital role played by the respiratory system. In 1681, the Italian physiologist and mathematician Giovanni Borelli was the first to attempt measuring the volume of inspired air in one single breath. Borelli used a device that entailed aspirating liquid up a cylindrical tube, which unfortunately did not account for the variable effort-related negative pressures, and therefore proved to be inaccurate. The British surgeon John Hutchinson published in 1846 his groundbreaking work on a device called the ‘spirometer’, which he used to systematically perform respiratory measurements. He then applied modern statistical methods that established ‘normal’ spirometric values, using previously unrecognized relationships between age, height, and the so-called ‘vital capacity’ [1–3]. The development of the spirometer accelerated tremendously the understanding of respiratory physiology. Similar key milestones in assessing airflow obstruction were Tiffeneau’s description of ‘circulating and captive air’ during exhalation [4] and Fry and Hyatt’s description of the ‘flow-volume curve’ [5], which helped us conceptualize the mechanics of pulmonary physiology.

To date, respiratory function evaluation focuses largely on volumes and airflows measured during forced expiratory maneuvers, without much consideration to inspiratory mechanics, leading some to call the oversight of inspiratory flow as ‘the neglected child of pulmonary diagnostics’ [6]. Furthermore, standards for test performance regarding the inspiratory limb of the flow-volume curve have also received little attention. Upper airway obstruction (UAO) is relatively uncommon compared with the lower airway obstruction encountered in those with asthma or chronic obstructive pulmonary disease (COPD). However, detecting UAO is critical because lesions may not manifest until very late, sometimes evolving quickly towards acute respiratory failure; and because treatment of UAO is dramatically different and occasionally curative.

Recently, Ibraheem et al. [7] described an alternative index to quantify the inspiratory loop, based on calculating the area of the inspiratory portion of the flow-volume curve (called AIN), and posited that it offers a potentially useful quantitative global measure of inspiratory mechanics, similar to the expiratory portion of the same curve (AEX) [8–16]. On the prospect that AIN might be useful in assessing UAO, normative reference standard values are needed in order to ascertain deviation from normal. Such reference standards are not, to our knowledge, currently available.

To address this gap, the current study characterizes AIN and AIN/AEX, as provided by one pulmonary function testing (PFT) digital system (Vyaire Medical, Chicago, Illinois), in healthy, asymptomatic, non-smoking individuals without respiratory symptoms or known pathology, from a large functional database at the Cleveland Clinic (Cleveland, Ohio).

## Methods

A descriptive analysis of PFTs was undertaken in a cohort of consecutive tests in one of the Cleveland Clinic PFT Laboratories between April 2019 and August 2020 (Cohort 0). The data set included 4,980 best-test, acceptable, single, pre- or post-bronchodilator normal spirometry sets (i.e., normal FEV_1_, FVC and FEV_1_/FVC based on reference standards according to the Global Lung Function Initiative [GLI, [17]]) from 4,846 individuals who met the following criteria: self-reported as never-smokers, without any known respiratory disease or respiratory complaints. Values above the lower limit of normal (LLN, the 5% percentile) by GLI equations were deemed normal [17]. To rigorously exclude subjects with a diagnosis of small airway disease, 134 tests were excluded from analyses because their FEF_75_ values were below the LLN by GLI equations [17].

In order to compare the descriptive statistics and the LLNs developed in Cohort 0, we compared the results in two other independent cohorts from the Cleveland Clinic PFT Laboratories (external validation):

- Cohort 1, comprising distinct PFTs completed between 2019 and 2020 (same time period) in the Cleveland Clinic PFT laboratory (n = 8,844 never-smokers, without any known respiratory disease or respiratory complaints, group non-overlapping with Cohort 0), and
- Cohort 2, comprising 21,253 spirometry studies completed between 2006 and 2007 (different time period) on 9,328 distinct adult patients who underwent same-day lung volume determinations by either body plethysmography or helium dilution method in the main campus Cleveland Clinic PFT Laboratory. Smoking status in these individuals was unknown. Among these tests, n = 4,376 were considered normal after exclusion of obstructive, restrictive, mixed ventilatory impairment patterns, as well as small airway disease.

Descriptive statistical analyses of available variables were performed. Categorical variables were summarized as frequencies or percentages. Continuous variables were characterized by mean (± Standard Deviation, Std Dev or SD), or as median and 25^th^-75^th^ interquartile range (IQR, expressed as the 25^th^ and 75^th^ percentile values), as most distributions were non-Gaussian. The Anderson-Darling A^2^ and Shapiro Wilk W tests were used for assessing goodness of fit for continuous variables. When usual transformations did not achieve fitting to normality by the above tests, we used non-parametric methods with native or log-transformed variables (e.g., Welch’s ANOVA, Wilcoxon rank score test or Kruskal-Wallis test, as appropriate) for comparisons. Between O’Brien[.5], Brown-Forsythe, Levene, Bartlett and 2-sided F tests used to compare for unequal variances, Levene test was assigned as the tie-breaker method when these tests were discordant.

Statistical analyses were performed using JMP Pro17 software (SAS Institute, Cary, NC, USA). Institutional research oversight approvals were obtained to conduct the study (Emory IRB# 00049576). Research data were last accessed on April 10^th^, 2024.

The analyses were performed in accordance with the relevant rules, guidelines and regulations (and regulatory approvals obtained from the respective institutional review boards). No informed consent was necessary, as these were data analyses of existing, publicly available databases.

## Results

### Cohort 0 (2019–2020, n = 4,846, all never-smokers)

The mean (±SD) age of study participants was 57.0 (±16.8) years; the median (25^th^-75^th^ IQR) was 59.7 (47.0–69.6) years; 21% were women (Table 1). By self-identified race or ethnicity, 71% were White, 17% Black, <1% Northeast Asian, <1% Southeast Asian, and 11% Other. All were lifetime never-smokers by self-report.

**Table 1:** Cohort 0 baseline characteristics.

	Mean (95% CI)	Std Dev	Percentile 5 (LLN)	Percentile 25 (Q1)	Percentile 50 (Median)	Percentile 75 (Q3)	Percentile 95 (ULN)
Age (years)	57 (56.55–57.50)	16.8	24.8	47	59.7	69.6	80.7
Height (m)	1.7 (1.73–1.73)	0.1	1.6	1.7	1.7	1.8	1.9
Weight (kg)	90.9 (90.26–91.49)	21.7	61.2	77.1	88.5	101.8	128
BMI (kg/m^2^)	30.3 (30.13–30.52)	6.8	21.7	25.6	29	33.7	42.5
FEV_1_ (L)	3.2 (3.13–3.18)	0.9	1.7	2.5	3.1	3.7	4.6
FEV_1_% pred	96.5 (96.15–96.80)	11.6	78.0	87.7	96	105.1	116.1
FVC (L)	4.1 (4.05–4.12)	1.1	2.3	3.3	4.1	4.9	5.8
FVC % pred	98.3 (97.95–98.61)	11.8	79.4	89.1	97.9	107	117.9
FEV_1_/FVC (%)	77.3 (77.08–77.42)	6.1	66.6	73	77.6	81.7	86.5
FEV_1_/FVC % pred	97.9 (97.77–98.13)	6.5	87.0	93.3	98.2	102.7	108.3

In cohort 0, 3,705 participants underwent ‘single’ spirometry battery tests (i.e., the required number of trials for quality assurance, and without assessment before and after bronchodilator, BD), and 1,141 participants had both pre- and post-BD tests.

The mean (±SD) and median (25^th^-75^th^ IQR) values of AIN were 16.0 (±9.1) and 14.7 (9.1–21.4) L^2^·sec^-1^, respectively (Table 2). Mean (±SD) best-trial measurements for the square root of AIN (Sqrt AIN, which ‘follows’ a Gaussian distribution) were 3.8 (±1.1) overall; 4.1 (±1.0) in men and 2.7 (±0.7) L·sec^-1/2^ in women. Mean (±SD) Sqrt AIN was 3.7 (1.8) in pre-BD and 3.8 (1.2) L·sec^-1/2^ in post-BD, respectively. In single tests, the mean (±SD) Sqrt AIN was 3.9 (±1.1) L·sec^-1/2^.

**Table 2:** AEX, AIN, AIN/AEX and their square root transformations in Cohort 0.

	Mean (95% CI)	Std Dev	Percentile 5 (LLN)	Percentile 25 (Q1)	Percentile 50 (Median)	Percentile 75 (Q3)	Percentile 95 (ULN)
**AIN (L** ^**2**^ **·sec** ^**-1**^ **)**	16.0 (15.79–16.31)	9.1	4	9.1	14.7	21.4	32.4
**F**	7.7	4.0	2.3	4.7	6.9	9.9	15.9
**M**	18.3	8.8	6.1	11.8	17.0	23.7	33.7
**Sqrt AIN (L·sec** ^**-0.5**^ **)**	3.8 (3.81–3.87)	1.1	2	3	3.8	4.6	5.7
**F**	2.7	0.7	1.5	2.2	2.6	3.1	4.0
**M**	4.1	1.0	2.5	3.4	4.1	4.9	5.8
**AEX (L** ^**2**^ **·sec** ^**-1**^ **)**	15.5 (15.24–15.67)	7.5	4.7	9.8	14.3	20.2	29.5
**F**	8.0	3.6	2.8	5.1	7.7	10.4	14.6
**M**	17.4	7.1	7.3	12.2	16.7	21.9	30.6
**Sqrt AEX (L·sec** ^**-0.5**^ **)**	3.8 (3.78–3.84)	1	2.2	3.1	3.8	4.5	5.4
**F**	2.8	0.6	1.7	2.3	2.8	3.2	3.8
**M**	4.1	0.8	2.7	3.5	4.1	4.7	5.5
**AIN/AEX (%)**	106.2 (105.14–107.32)	38.7	51.6	77.7	101.7	129.4	174.7
**F**	99.6	37.9	47.1	70.6	96.0	122.0	167.2
**M**	108.0	38.7	53.1	79.4	104.1	131.6	177.0
**Sqrt AIN/Sqrt AEX (%)**	101.3 (100.82–101.88)	18.7	71.8	88.1	100.9	113.7	132.2
**F**	98.0	18.8	68.6	84.0	98.0	110.4	129.3
**M**	102.2	18.6	72.9	89.1	102.0	114.7	133.0

**Fig 1:**
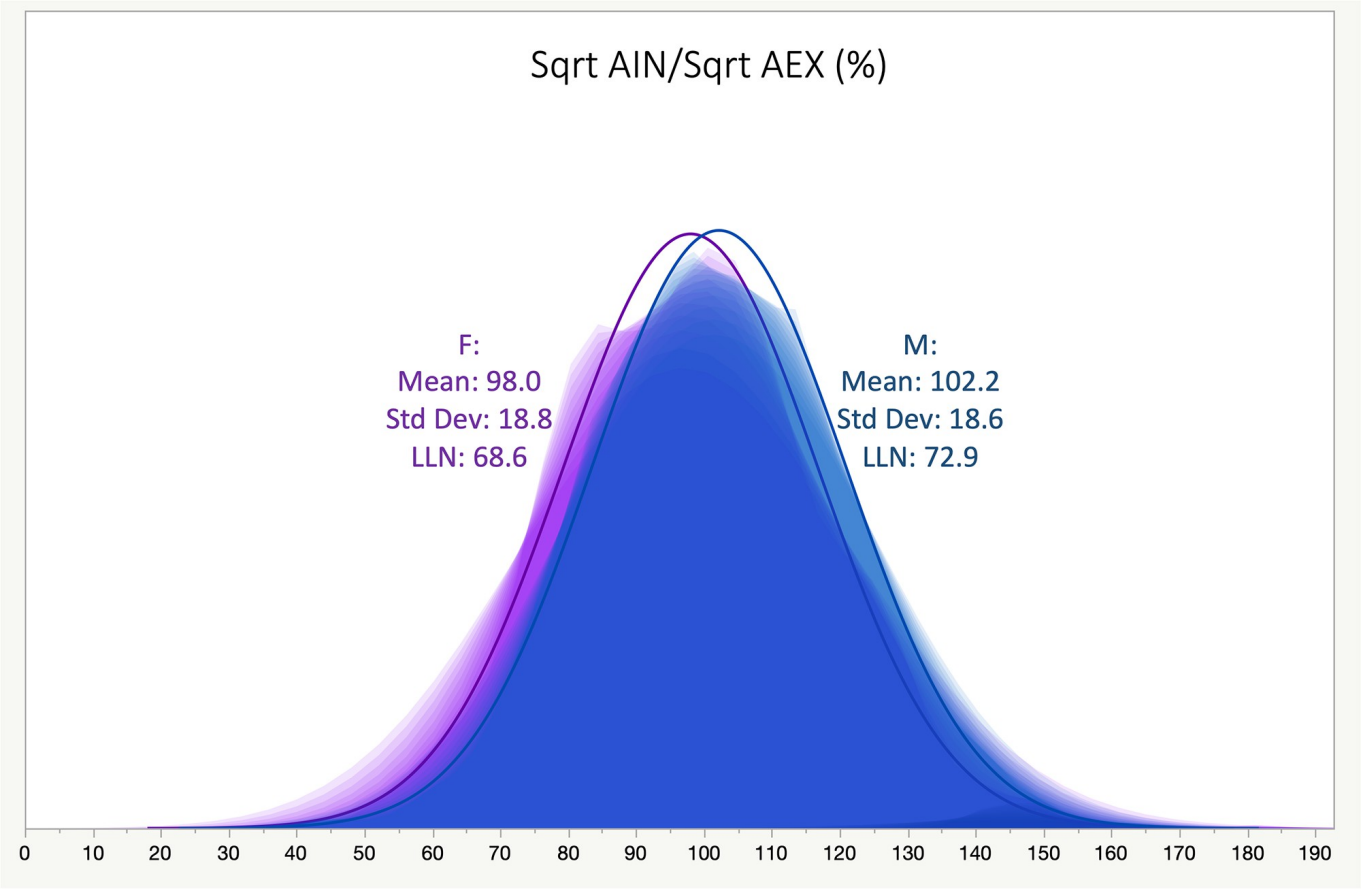
Square root AIN x 100/Square root AEX (%) distributions in cohort 0 by gender (M = Males, F = Females). LLN: Lower Limit of Normal (lowest 5% percentile); Std Dev: Standard Deviation.

**Fig 2:**
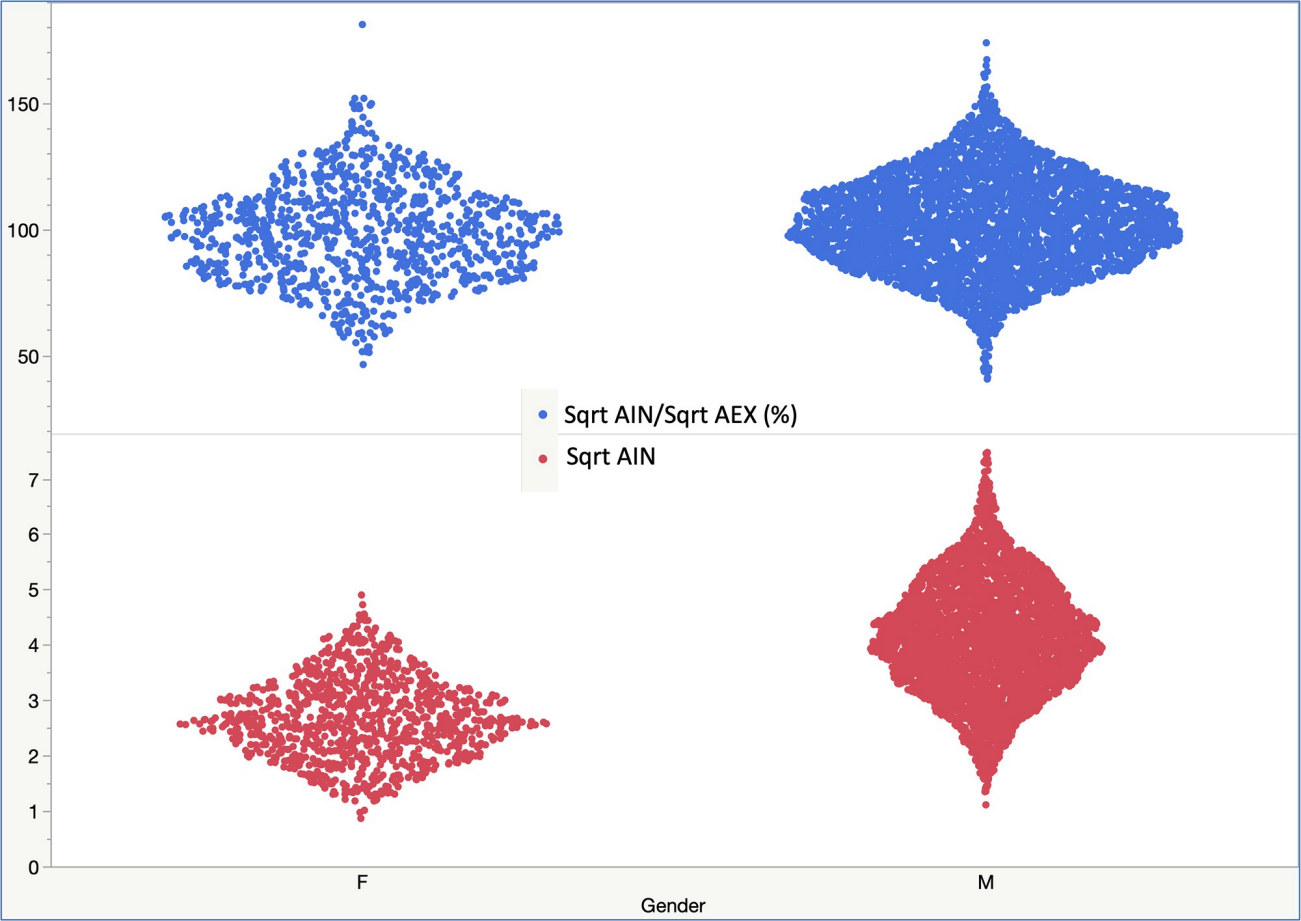
Square root AIN (Sqrt AIN) and Square root AIN x 100/Square root AEX [Sqrt AIN/Sqrt AEX (%)] distributions in cohort 0 by gender (M = Males, F = Females). LLN: Lower Limit of Normal (lowest 5% percentile).

**Fig 3:**
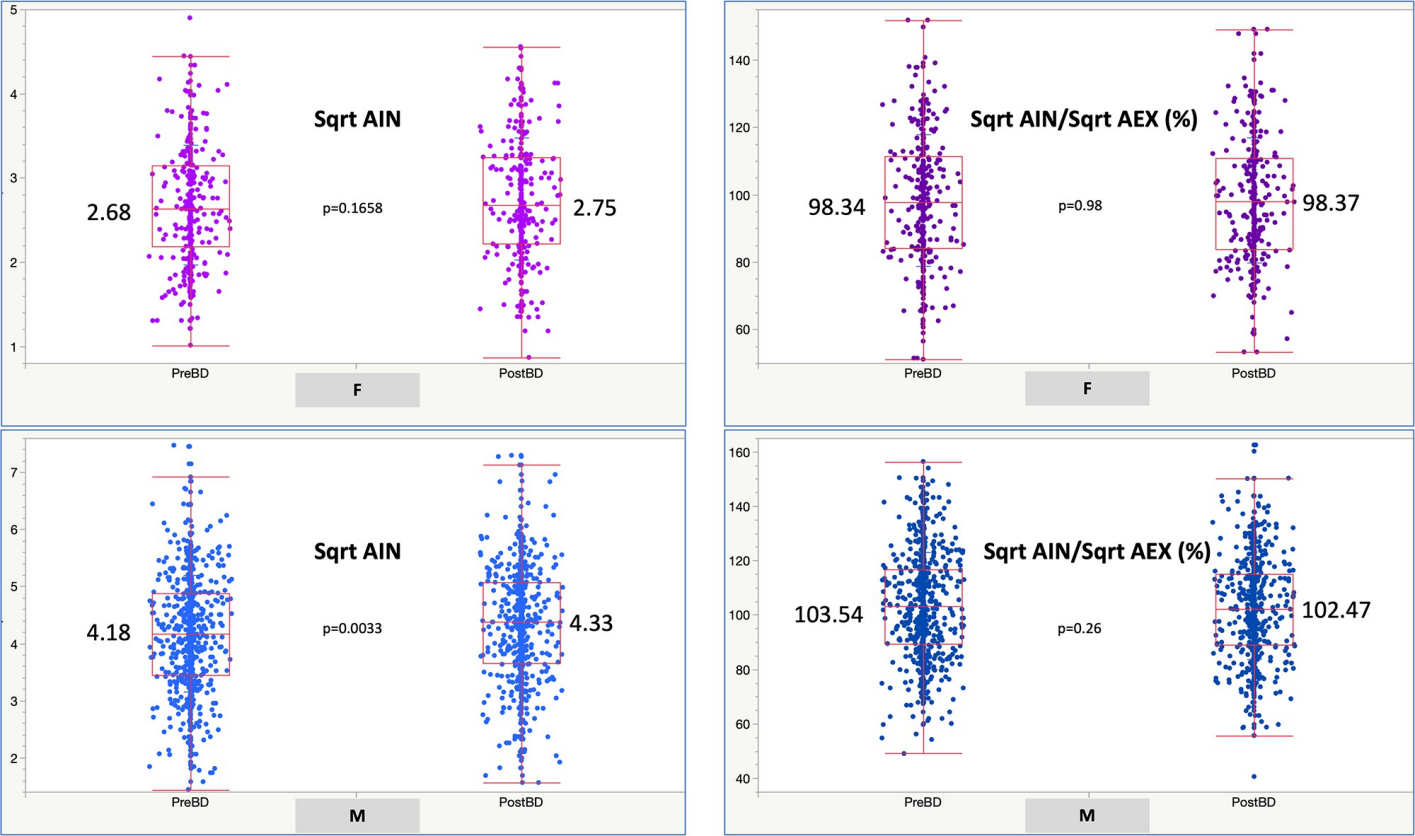
Square root AIN (Sqrt AIN) pre-BD and post BD in cohort 0 by gender (M = Males, F = Females). BD: bronchodilator.

**Fig 4:**
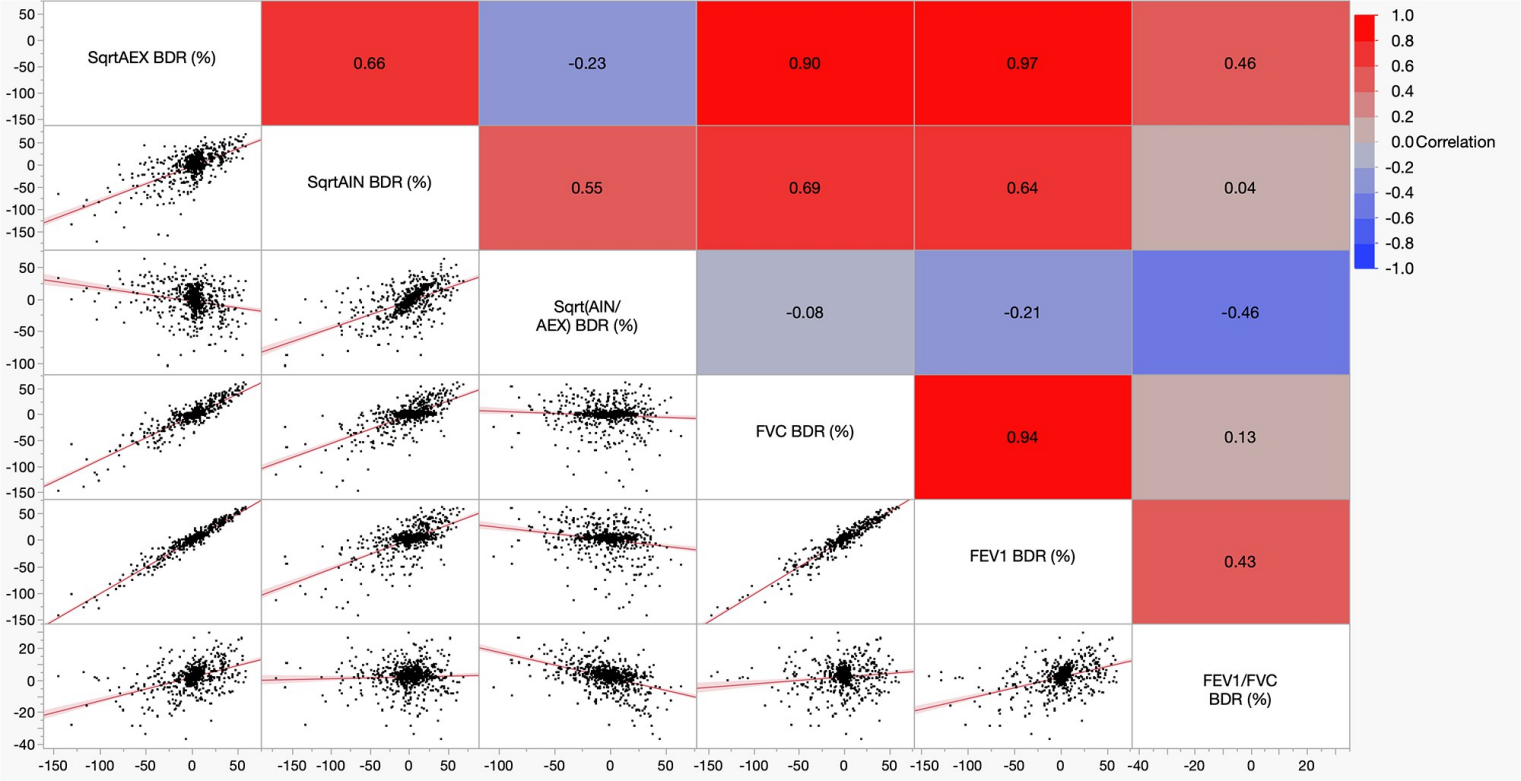
Multivariate analysis—Scatterplot matrix of different physiologic parameters’ bronchodilator responsiveness (BDR).

The mean (95% Confidence Interval, CI) ±SD of the square root of AIN to square root of AEX times 100 [Sqrt AIN/Sqrt AEX, (%)] was 101.3 (CI: 100.8–101.9) ±18.7; the lower 5^th^ percentile was 71.8%. Stratified by gender, the mean (±SD), median (25^th^-75^th^ interquartile range, IQR) and lowest 5% percentile (LLN) of the Sqrt AIN/Sqrt AEX (%) was 98.0 (±18.7), 97.9 (84.0–110.5), and 68.6% in women, and 102.2 (±18.6), 102.0 (89.1–114.7) and 72.9% in men, respectively (p<0.0001, in-between group comparison by Tukey-Kramer HSD test); consequently, LLNs were computed by gender (male or female, Figs 1 and 2). Similarly, the mean Sqrt AIN/Sqrt AEX (%) was 102.7, 98.4, 98.2, 95.3, and 97.9% in Whites, Blacks, Northeast Asians, Southeast Asians, or in Other categories, respectively (p<0.0001, in-between group comparisons by rank sums Kruskal-Wallis test). Values for the variance explained in correlations between Sqrt AIN/Sqrt AEX (%) and age, height, weight, and body mass index, were R^2^ 0.02, 0.006, 0.006 and 0.02, respectively. The mean (±SD) pre- and post-BD test values for Sqrt AIN/Sqrt AEX (%) were 101.9 (±19.6) % in pre-BD and 101.12 (±18.7) % in post-BD (Fig 3). In single tests, the mean (±SD) Sqrt AIN/Sqrt AEX (%) was 101.7 (±18.3) %. Mean (CI) ±SD, median (25^th^-75^th^ IQR) and LLN for the SqrtAIN/SqrtAEX (%) bronchodilator responsiveness (BDR, i.e., post-BD minus pre-BD divided by pre-BD, percent) were -3.0 (CI: -4.3 –-1.8) ±21.6, -2 (-12.9–10.4) and -44.1% (see in Table 3 and Fig 4 the other physiologic parameters’ BDR and their correlation, respectively).

**Table 3:** FEV_1_, FVC, FEV_1_/FVC ratio, SqrtAIN, SqrtAEX and SqrtAIN/AEX bronchodilator responsiveness (BDR) in Cohort 0.

	Mean (95% CI)	Std Dev	Percentile 5 (LLN)	Percentile 25 (Q1)	Percentile 50 (Median)	Percentile 75 (Q3)	Percentile 95 (ULN)
FEV_1_ BDR (%)	0.8 (-0.61–2.28)	24.6	-49.9	-2.0	2.9	8.1	38.4
F	-2.0	28.8	-59.1	-1.3	2.8	7.6	38.4
M	2.2	22.1	-38.0	-2.2	2.9	9.2	38.8
FVC BDR (%)	-1.1 (0.21–0.67)	22.4	-44.9	-4.3	0.2	4.9	36.0
F	-4.1	27.4	-55.8	-5.3	-0.4	4.8	35.2
M	0.4	19.4	-36.4	-3.9	0.5	4.9	36.3
FEV_1_/FVC BDR (%)	2.1 (1.69–2.58)	7.5	-12.5	-0.2	2.7	5.6	13.3
F	2.1	6.5	-12.1	-0.1	2.7	5.6	12.0
M	2.1	8.0	-12.6	-0.3	2.7	5.6	14.4
SqrtAIN BDR (%)	-0.8 (-2.48–0.88)	28.5	-59.0	-8.8	2.0	14.1	39.2
F	-3.4	32.9	-74.5	-8.8	2.0	14.1	41.3
M	0.5	26.0	-40.8	-8.5	1.9	14.2	38.0
SqrtAEX BDR (%)	1.0 (-0.35–2.44)	23.7	-43.3	-2.5	3.4	9.1	35.3
F	-1.5	27.0	-57.5	-2.9	3.6	8.8	33.5
M	2.3	21.9	-40.1	-2.3	3.3	9.7	36.5
SqrtAIN/SqrtAEX BDR (%)	-3.0 (-4.31–-1.77)	21.6	-44.1	-12.9	-2.0	10.4	30.3
F	-2.6	20.5	-38.6	-12.0	-1.6	10.1	29.3
M	-3.3	22.2	-46.3	-13.1	-2.1	10.5	30.7

### Cohort 1 (2019–2020, n = 8,844, all never-smokers)

The mean (±SD) age of study participants was 56.9 (±16.5) years; median (25^th^-75^th^ IQR) was 59.7 (46.5–69.2) years; 56% were women; by self-identified race and/or ethnicity, 87% were White, 13% Black, <1% Northeast Asian, Southeast Asian or Other. The mean (±SD) and median (25^th^-75^th^ IQR) values of AIN were 16.7 (±7.8) and 8.8 (4.9–14.6) L^2^·sec^-1^, respectively.

The mean (95% CI) and standard deviation of Sqrt AIN/Sqrt AEX (%) were 107.0 (95% CI: 106.5–107.6) and 26.0%, respectively. Mean Sqrt AIN/Sqrt AEX (%) was 108.4% in men and 105.9% in women.

The percentage of Sqrt AIN/Sqrt AEX (%) <LLN (as determined in cohort 0) was 4.5%.

### Cohort 2 (2006–2007, n = 4,376 normal, unknown smoking status)

The mean (±SD) age of study participants was 56.4 (±14.0) years; the median (25^th^-75^th^ IQR) was 57 (47–67) years; 48% were women; by self-identified race and/or ethnicity, 86% were White, 14% Black, and <1% Northeast Asian, Southeast Asian, or Other.

Among those with a normal ventilatory pattern (i.e., normal spirometry and lung volumes), mean (±SD) and median (25^th^-75^th^ IQR) values of AIN were 11.9 (±7.7) and 10.3 (6.6–14.8) L^2^·sec^-1^, respectively.

The mean (95% CI) and SD values of Sqrt AIN/Sqrt AEX (%) were 101.5 (95% CI: 99.2–103.8) % and 17.5%, respectively. The mean Sqrt AIN/Sqrt AEX (%) value was 102.7% in men and 100.5% in women.

The percentage of Sqrt AIN/Sqrt AEX (%) <LLN (as determined in cohort 0) among those characterized as having normal function was 1.8%.

When compared by different, observed ventilatory defects, the mean Sqrt AIN/Sqrt AEX (%) was 183.8%, 140.3%, 114.4%, 106.3%, and 97.3% in obstruction, mixed ventilatory defects, small airway disease, restriction, and with normal patterns, respectively. The degree of ‘separation’ was statistically significant between obstruction and all the other categories; however, the measurement did not differentiate between restriction, small airway disease, and normal patterns.

In all 3 cohorts, Sqrt AIN and Sqrt AIN/Sqrt AEX (%) were significantly higher in men vs women (p<0.0001, Wilcoxon or Tukey Kramer HSD test).

In multivariate models, the functional parameter Sqrt AIN was moderately influenced by gender, race/ethnicity, age, and height (similar to other spirometric measurements). As such, the variance explained by these variables (R^2^) using linear and generalized regression (lasso, double lasso, elastic net, ridge) models in all three cohorts was between 0.36 and 0.56 (Table 4). Very little of the Sqrt AIN/Sqrt AEX (%) variance was explained by age, height or gender (R^2^<0.04, unlike other spirometric measurements).

**Table 4:** Models of Sqrt AIN and Sqrt AIN/Sqrt AEX (%) based on age, height, gender and race, and age, height, and gender, respectively, using regular regression (standard least squares) and generalized regression using lasso, double lasso or elastic net (regular or with adaptive features) or ridge regression techniques, with 30% random holdback validation.

Estimation Method of Sqrt AIN based on age, height, gender, and race/ethnicity	Validation Model R^2^			Estimation Method of Sqrt AIN/Sqrt AEX (%) based on age, height, and gender	Validation Model R^2^		
	Cohort 0	Cohort 1	Cohort 2		Cohort 0	Cohort 1	Cohort 2
**Standard Least Squares**	0.54	0.54	0.39		0.03	0.03	0.01
**Lasso**	0.54	0.55	0.39		0.02	0.02	0.01
**Adaptive Lasso**	0.56	0.53	0.34		0.03	0.03	0.01
**Double Lasso**	0.53	0.52	0.36		0.03	0.04	0.01
**Adaptive Double Lasso**	0.52	0.55	0.46		0.03	0.04	0.01
**Elastic Net**	0.55	0.53	0.40		0.03	0.02	0.02
**Adaptive Elastic Net**	0.51	0.56	0.43		0.04	0.03	0.01
**Ridge**	0.56	0.52	0.36		0.03	0.02	0.01

## Discussion

In this study, we derived normative values (LLN) for the digitally computed area under inspiratory flow-volume curve (AIN) in normal, never-smoker individuals. This determination sets the stage for further study to assess the diagnostic utility of this spirometric parameter (or the square root of the AIN divided by square root of AEX times 100) in diagnosing upper airway obstruction and differentiating among its many potential causes, both intra- and extra-thoracic.

Historically, respiratory function evaluation had focused mainly on forced maximal expiratory volumes and airflows [6]. This may have been due, at least in part, to the fact that functional assessment is less sensitive at higher lung volumes, that the inspiratory resistance to airflow does not factor in the (passive) elastic recoil of the respiratory system, and that the measurement is more influenced by the magnitude of the (active) respiratory effort. Furthermore, performance standards for alternative respiratory measurements, such as the inspiratory limb of the flow-volume curve, the amount of air inhaled during expiratory lung volume determinations, and the attained total lung capacity for diffusing capacity measurements had been similarly overlooked for decades.

The two most recent documents on standardization and technical standards for spirometry issued by the American Thoracic Society (ATS) in conjunction with the European Respiratory Society (ERS) attempt to make such corrections by emphasizing the importance of coaching to a full inhalation before and after a forced exhalation, and using forced inspiratory vital capacity as one of the acceptability criteria needed for a valid effort [18, 19]. Given the renewed attention to the inspiratory portion of the flow-volume curve, there is an emerging need to characterize and operationalize these new parameters for clinical application. For example, peak inspiratory flow (PIF) measurement has proven to be useful in guiding specific bronchodilator use in asthma and COPD. Forced inspiratory flow at 50% (FIF_50_), used in isolation or in conjunction with its forced expiratory equivalent (FEF_50_), has also been found useful as an indicator of upper airway obstruction. Unfortunately, some of these indices (e.g., the ratio FEF_50_/FIF_50_) are limited by poor reliability or high variability and poor diagnostic performance [20, 21].

Upper airway conditions such as tracheal stenosis, tracheobronchomalacia, laryngeal or other upper respiratory tumors, are relatively uncommon compared with lower airway obstruction seen, for example, in asthma, COPD or small airway disease. Recognizing upper airway obstruction (UAO) is critical because it may manifest clinically only in advanced stages and has the potential to evolve quickly towards acute respiratory failure. Also, the treatment for UAO differs greatly from that for lower airways obstructive disorders and may be occasionally curative.

Diagnosing UAO with spirometry may be elusive because, in the early stages, central airway obstruction may not lead to decreased FEV_1_ or FVC, while PEF becomes subtly and progressively reduced. Studies of maximal inspiratory flow-volume curves showed that increased resistance in the upper airways leads to a reduction of inspiratory flow rates at all lung volumes, but that the contour of the curve still resembles that of the normal pattern [22]. Thus, diagnosis may be similarly elusive [21]. Some indices (e.g., PEF, FEV_1_/PEF, FEV_1_/FIV_1_, FIF_50_, FIF_50_/FEF_50_, etc.) may suggest the diagnosis of UAO, or could aid in distinguishing intrathoracic vs extrathoracic airway obstruction [19, 23], but they lack the necessary precision for diagnosing UAO.

Ibraheem et al. [7] noted recently that existing, publicly available raw spirometry datasets do not include inspiratory flow-volume data; as such, they obtained *de novo* a new raw dataset on 129 healthy adult research participants. The new spirometric measurement was based on the calculated area under the inspiratory portion of the flow-volume curve (called AIN), and was derived by using the trapezoidal numerical integration method, similar to the derivation of the AEX, as published by us and others before [9, 10, 13]. Ibraheem et al. posited that this alternative measurement offers a potentially useful quantitative global measure of inspiratory mechanics, similar to the expiratory portion of the same curve (AEX) [8–16].

Our current study analyzed the distribution of AIN, AEX, AIN/AEX, square root of AIN, square root of AEX, and the ratio of square root of AIN by square root of AEX times 100 (square root transformations were done to ‘normalize’ distributions) and identified their lower limits of normal (LLN). While this is, to our knowledge, the first time such values have been proposed, it is recognized that full utility will require both further investigation of the diagnostic value of AIN for UAO, and validation of these normative results in other, large, independent populations of normal individuals. The Sqrt AIN was mainly determined by gender, age and total body height; nevertheless, the percent of linear or generalized regression (lasso, elastic net, ridge, double lasso method, etc.) models’ variance explained by these variables was <52% (results not shown here). The Sqrt AIN/Sqrt AEX (%) was largely independent of age, total body height or weight, making it a potentially attractive parameter for diagnosing UAO. Since the parameter differed by gender and followed a normal distribution, the LLN developed this way and using the normal distribution’s 5% threshold as the LLN is likely valid.

The current study has several strengths. First, to our knowledge, this is the first attempt at computing normative values for AIN. Second, the computations (made available by the PFT vendor’s software) derive from large cohorts of spirometric tests from healthy adults. Third, the LLNs of these parameters were validated in two additional cohorts, i.e., one of never-smokers with no known history of respiratory disease or symptoms in whom pre-, post- bronchodilator or single spirometry tests were analyzed, and another of consecutive patients from a different time period, who also had same-day lung volume determinations (the latter ensuring that we can compare the normative data from cohort 0 with those who had normal lung function by current GLI criteria, including some to exclude small airway diseases).

Several limitations of the current study also warrant mention. First, all cohorts analyzed included only adult participants (age ≥18 years); similar analyses in children are needed. Second, we acknowledge that these were retrospective analyses done on cohorts of PFTs without detailed or curated histories or known indications for the tests performed; as such, one cannot absolutely exclude the possibility that some subjects may have had mild respiratory impairments or comorbidities that could have affected the measured volumes and flows. Third, the derived LLNs need to be validated in larger, independent cohorts of patients undergoing PFTs in different centers, from different geographies, and with different ethnic distributions. Fourth, the smoking status of subjects was based on self-report, without objective documentation (e.g., cotinine levels, etc.). Fifth, further analysis of within-subject reproducibility (variance) of these indices is needed. Sixth, the population tested had weights and BMIs conceivably higher than a traditional lean, healthy cohort, but one can easily argue that it represents in fact the current population, a consequence of historical trends in the western society, of progressively higher prevalence of obesity and overweight. Finally, the current analysis had not referenced the latest, race-neutral GLI normative equations for PFTs, as has been recently advocated [24, 25]. In the near future, we do plan to re-analyze these data with respect to the newly proposed reference standards.

## Conclusion

The new functional parameters using AIN provide normative standards that, with further investigation, offer promise to enhance the diagnostic approach to UAO, especially because some of these parameters appear to be independent of age, race, height and weight. The quantitative assessment of the relationship between AIN and AEX (area under the expiratory flow-volume curve) may also prove to be useful in assessing conditions affecting expiratory flow (e.g., asthma and chronic obstructive pulmonary disease). Further validation of the derived lower limits of normal of the (square root) AIN and AIN/AEX is clearly needed, with the understanding that confirmation of these values should prompt manufacturers of pulmonary function testing equipment to provide quantitative measurements derived from the inspiratory flow-volume curves in their equipment software (e.g., AIN, FIF_50_, FIF_50_/FEF_50_, FIV_1_/FEV_1_, etc.).

## Abbreviations

AEX: Area Under Expiratory Curve
AIN: Area Under Inspiratory Curve
ATS: American Thoracic Society
BD: Bronchodilator
BMI: Body Mass Index
CI: Confidence Interval
COPD: Chronic Obstructive Pulmonary Disease
ERS: European Respiratory Society
FEF_50_: Forced Expiratory Flow at 50% of Forced Vital Capacity
FEV_1_: Forced Expiratory Volume in 1 second
FIF_50_: Forced Inspiratory Flow at 50% of Forced Vital Capacity
FIV_1_: Forced Inspiratory Volume in 1 second
FVC: Forced Vital Capacity
GLI: Global Lung Initiative
HSD: Tukey-Kramer’s Honest Significant Differences
IQR: Inter-Quartile Range
IRB: Institutional Review Board
LLN: Lower Limit of Normal
PEF: Peak Expiratory Flow
PFT: Pulmonary Function Testing
Sqrt: Square Root Function
Std Dev or SD: Standard Deviation
UAO: Upper Airway Obstruction
